# An Open-Label, Head to Head Comparison Study between Prucalopride and Lactulose for Clozapine Induced Constipation in Patients with Treatment Resistant Schizophrenia

**DOI:** 10.3390/healthcare8040533

**Published:** 2020-12-03

**Authors:** Ishwary Damodaran, Koh Ong Hui, Amer Siddiq Amer Nordin, Anne Yee, Jesjeet Singh Gill, Benedict Francis, Fatin Liyana Azhar, Ahmad Hatim Sulaiman

**Affiliations:** 1 Hospital Bentong, Jalan Tras, Bentong 28700, Malaysia; ishwari@moh.gov.my; 2 Department of Psychological Medicine, Faculty of Medicine, University of Malaya, Kuala Lumpur 50603, Malaysia; ohkoh@um.edu.my (K.O.H.); amersiddiq@um.edu.my (A.S.A.N.); annyee17@um.edu.my (A.Y.); jesjeet@um.edu.my (J.S.G.); fatin.azhar@um.edu.my (F.L.A.); 3 Department of Psychological Medicine, University Malaya Medical Centre, Kuala Lumpur 59100, Malaysia; benedict@ummc.edu.my

**Keywords:** clozapine induced constipation, prucalopride, lactulose, efficacy, safety

## Abstract

Clozapine remains as the gold standard for the management of treatment resistant schizophrenia. Nevertheless, mortality and morbidity associated with Clozapine is partly contributed by its adverse effect of constipation in view of its prominent anticholinergic properties. Despite the evidence that approximately 60% of patients on Clozapine will experience constipation, there is no proper guideline as to the best laxative in the treatment of Clozapine induced constipation. Hence this study was conducted to evaluate the efficacy and safety of Prucalopride and Lactulose in the treatment of Clozapine induced constipation. This was a four week, prospective, open-label head to head comparison study between Prucalopride and Lactulose in the treatment of Clozapine induced constipation. Male and female patients on Clozapine between the age of 18–60 with an established diagnosis of treatment resistant schizophrenia with ≤2 spontaneous complete bowel movement per week were recruited in this study. Eligible patients were assigned into two groups. Patients received Prucalopride 2 mg once daily or Lactulose 10 g once daily for four weeks. Efficacy was analyzed in 58 patients. The proportion of patient with ≥3 spontaneous complete bowel movement (SCBM) was higher in the Prucalopride 2 mg group, reaching significance at Week 4 with *p*-value of (*p* = 0.029). The proportion of patient with ≥3 SCBM at Week 1 was 71.4% in the Prucalopride 2 mg group and 60% in the Lactulose 10 g group. The proportion of patient with ≥3 SCBM at Week 4 was 85.7% in the Prucalopride 2 mg group and the proportion remained at 60% in the Lactulose 10 g group. The improvement in the dissatisfaction and treatment satisfaction subscales of the patient assessment of constipation—quality of life (PAC-QOL) were higher in the Prucalopride 2 mg group compared to the Lactulose 10 g group. The common adverse events associated with Prucalopride 2 mg were abdominal pain and loose stools which was transient and subsided within a few days. Over four weeks, in this population of patients with Clozapine induced constipation, Prucalopride 2 mg significantly improved the bowel movement and it was safe.

## 1. Introduction

Clozapine remains as the mainstay evidence based pharmacotherapy to treat patients with treatment resistant Schizophrenia. It is the most effective antipsychotic for treatment resistant Schizophrenia [1,2,3]. Clozapine plasma concentration of more than 350 ng/mL is recommended for therapeutic response [4]. Unfortunately, Clozapine causes intestinal hypomotility. Constipation is reported in about 60% of patients on Clozapine [5]. Inadequate treatment of constipation will result in intestinal obstruction, paralytic ileus and contributes to mortality by antipsychotics [6,7]. Proposed mechanisms for gastrointestinal slowing with clozapine include its significant anticholinergic effects, as well as 5-HT3 antagonism.

A cross sectional study measuring colonic motility in patients with Clozapine and other antipsychotic medication was conducted and the median colonic transit time was four fold higher in patients on Clozapine compared to other antipsychotic medication. A total of 80% of those prescribed with Clozapine showed reduced colonic motility than those on other antipsychotic medication. The other antipsychotics were Aripiprazole, Risperidone, Paliperidone, Zuclopenthixol, Olanzapine and Haloperidol. The median colonic transit time was 104.5 h for patients on Clozapine and 23 h for those on other antipsychotics [8].

Recent researches also indicate mortality due to complications of clozapine induced constipation is about 10–12 per 10,000 [9,10]. This mortality rate is almost three times more than the frequently reported complication due to agranulocytosis [11].

There are inadequate good quality trials comparing the use of laxatives in antipsychotics induced constipation let alone in Clozapine induced constipation. There have not been proper clinical trials to suggest the effectiveness of conventional laxatives such as lactulose, polyethylene glycol, stool softeners or other available options such as Prucalopride and Linaclotide to treat antipsychotics induced constipation.

This study was conducted in view of there being minimal literature available for this distressing adverse effect of Clozapine.

Assertive evaluation and early detection of clozapine induced gastrointestinal hypomotility would reduce the mortality rate associated to this adverse effect.

The aim of this study was to evaluate the efficacy and safety of Prucalopride 2 mg and Lactulose 10 g in the treatment of Clozapine induced constipation. The primary objective of this study was to compare the proportion of patients with ≥3 spontaneous, complete bowel movements (SCBM) at Week 1 and Week 4 in both the treatment groups, respectively. The secondary objectives were to compare the quality of life in patients on Prucalopride 2 mg and Lactulose 10 g using the patient assessment of constipation—quality of life (PAC-QOL) questionnaire prior to treatment initiation and upon completion of the study intervention at Week 4. Another secondary objective was to assess and evaluate the presence of adverse events in both the study treatment.

## 2. Materials and Methods

This was an open label, head to head comparison study, between Prucalopride 2 mg and Lactulose 10 g for Clozapine induced constipation in patients with treatment resistant schizophrenia, conducted over duration of 4 weeks.

This study was carried out at University Malaya Medical Center (UMMC) which is situated in Kuala Lumpur, in the Federal Territory of Malaysia. The population of this area consists of mostly Chinese, Malay and Indian ethnicities. Since its establishment in 1965, UMMC has been one of the country’s most prominent tertiary referral centers. This center not only provides inpatient and outpatient services, it is also well known for its extensive clinical work and researches. The Department of Psychological Medicine was founded in the same year as UMMC in 1965, soon after the establishment of the medical faculty of the University of Malaya in 1964.

### 2.1. Inclusion and Exclusion Criteria

Male and female outpatients between the ages of 18–60 on Clozapine for treatment resistant schizophrenia were eligible to participate in this study. Participants who were stable on the same dose of Clozapine for at least 6 months were ensured. Participants had to be on a fixed and regular dosage of Clozapine during the trial without any changes in dosage or addition of a different medication apart from their existing medication. The participants had to fulfil the criteria of constipation according to ROME III criteria of constipation: 2 or fewer spontaneous bowel movements per week that result in a feeling of incomplete evacuation with one or more of the below: hard stools, sensation of incomplete evacuation or straining at defecation.

There were several exclusion criteria for the study which includes participants who are unable to give informed consent, participants with large bowel disorders (i.e., irritable bowel syndrome, Crohn’s disease, megacolon). Apart from that, participants with clinically uncontrolled cardiac diseases, kidney, liver and lung diseases as well as participants with clinically significant abnormal laboratory values including renal profile were excluded from this study.

### 2.2. Study Intervention

Participants were allocated to one of the two groups using systematic sampling. Study participants were allocated according to their call number whereby study participants with odd numbers were allocated into the Prucalopride 2 mg group and study participants with even numbers were allocated into the Lactulose 10 g group. Balancing was ensured to make sure equal number of patients entered each treatment group. There was a washout period of 48 h in which no laxatives were given prior to initiation of either Prucalopride 2 mg or Lactulose 10 g. Enema was prescribed for the subjects as rescue medication if participants had not had a bowel movement similar to the frequency which was documented at baseline during the subsequent weeks in the study. The participants were allowed to be on other antipsychotics besides Clozapine as almost half of the population of interest were on a combination treatment of antipsychotics for their diagnosis of treatment resistant schizophrenia. Additionally, the use of benzodiazepines and benzhexol were allowed as they were also commonly prescribed in this population. The use of antidepressants was permitted if the participant had been on a stable dose for 6 months and above.

### 2.3. Efficacy Evaluation

Information on bowel movement which includes frequency, consistency, straining and sensation of complete evacuation were obtained and recorded during the baseline visit. Study participants were instructed to keep a record of their bowel movement throughout the 4 weeks intervention period. These records were assessed during the individual week and over the 4 weeks period of assessment.

The primary endpoint was the proportion of participants having on average ≥3 spontaneous complete bowel movement at Week 1 and at the end of Week 4. The secondary endpoint was to compare the quality of life associated to constipation in participant treated with Prucalopride 2 mg and Lactulose 10 g using the patient assessment of constipation—quality of life (PAC-QOL) questionnaire. The patient assessment of constipation—quality of life questionnaire (PAC-QOL) is a validated questionnaire which consists of 28 questions and is subdivided into four subscales. The first 3 subscales are the dissatisfaction subscales: physical discomfort (4 questions), psychosocial discomfort (8 questions), worries and concerns (11 questions) and the fourth subscale is the treatment satisfaction subscale (5 questions) [12]. Permission was obtained from Mapi Research trust in order to use this questionnaire.

The scoring is done with a five-point Likert scale, scoring between 0 and 4. Zero indicates “none at all” and 4 indicates “extremely”. A lower score indicated a better quality of life. Scores of items 18 and 25 to 28 are reversed. Scale scores are then computed as the mean values of the scale. The treatment outcome analysis for PAC-QOL is a validated response whereby increment by 1 point in the score from the initial assessment was taken as the response level to evaluate the differences between treatment groups. The PAC-QOL was assessed prior to initiating the treatment and upon completion of the study intervention at Week 4.

### 2.4. Safety Evaluation

Presence of adverse events were evaluated and recorded during the individual week up until the end of the intervention. Patients on Clozapine are required for regular blood taking during their follow up in view of risk of agranulocytosis with Clozapine use. Therefore, baseline blood parameters which include complete blood count and renal profile were obtained as per protocol for the study participants to ensure there were no abnormal laboratory values. Apart from that, vital signs were recorded during the baseline visit and follow up evaluation. Additionally, past medical history and past surgical history were also obtained prior to initiation of the treatment.

### 2.5. Statistical Analysis

The estimation of sample size for this pilot study was determined using a sample size calculation software. It was based on assumed response rates for the primary endpoint. The value of 80% was set as the power of the study. A 95% confidence interval level was used for the significant value and α was set at 0.05. The proportion for Prucalopride was determined at 69% and the proportion for Lactulose was determined at 31%. The ratio of sample size, exposed to unexposed is 1:1. Therefore the required sample size was 28 patients per treatment group. Socio-demographic data as well as the outcome variables was summarized using descriptive analysis. Mean, median and standard deviation were used to describe the outcome of the continuous variables while percentage and frequency were used to describe the results of categorical variables. A test of normality was performed to decide the distribution of the continuous outcome variables. The chi square test was used to determine the association between categorical variables. Mann–Whitney U test was used to determine the difference of the outcome variables between the intervention groups, the group receiving Lactulose 10 g and Prucalopride 2 mg. All the relevant statistical analysis was analyzed using the Statistical Package for Social Science (SPSS) Version 23.0 and *p*-values of less than 0.05 were considered to be significant.

## 3. Results

**Figure 1:**
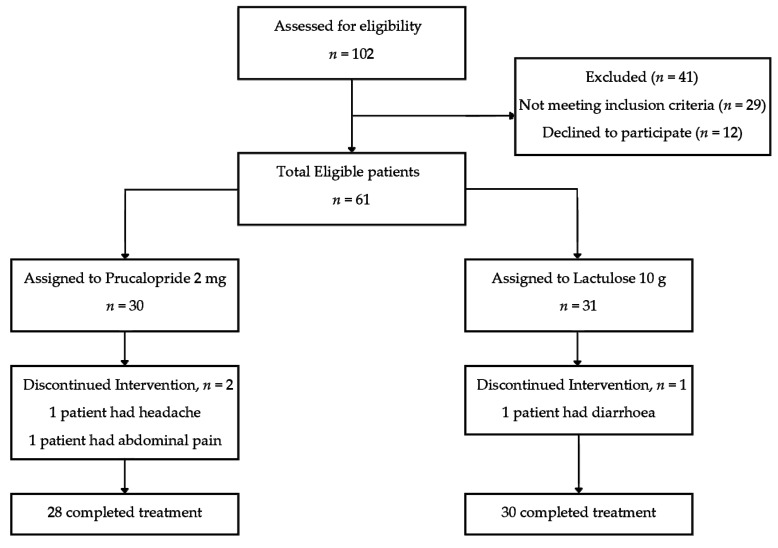
Disposition of patients in the open label, head to head comparison study between Prucalopride and Lactulose for Clozapine induced constipation in patients with treatment resistant schizophrenia.

A total of 102 patients were approached to be recruited in this study (Figure 1). A total of 41 patients were not included in this study for several reasons. A total of 29 of them did not meet the inclusion criteria while 12 patients declined to participate in the study. Therefore, there were a total of 61 eligible patients who were recruited in this study. From the total of 61 patients who were recruited in this study, 30 were allocated to the Prucalopride 2 mg group and 31 were allocated to the Lactulose 10 g group.

There were drop outs in both the treatment group during the first week due to presence of adverse events. There were 2 drop outs from the Prucalopride 2 mg group whereby 1 patient developed headache and 1 patient developed abdominal pain. There was 1 drop out from the Lactulose 10 g group whereby the patient developed diarrhea during the first week. These patients were not included in the evaluation as they did not complete the study. A total of 28 patients completed the study from the Prucalopride 2 mg group and 30 patients completed the study form the Lactulose 10 g group.

### 3.1. Socio-Demographic and Patient Characteristics Profile

An overview of the socio demographic profile of patients recruited into this study is presented in Table 1. Overall, for the socio- demographic profile, there were no statistically significant differences between the groups in terms of age, gender, ethnicity, marital status and employment using Chi square test for the categorical data or the Independent *t*-test for the continuous data.

The majority of the patients were from the Chinese ethnicity in both Prucalopride and Lactulose group. There were 71.4% (*n* = 20) and 63.3% (*n* = 10) of Chinese participants respectively. There were 14.3% (*n* = 4) and 23.3% (*n* = 7) Malay participants respectively and (14.3%) and (13.3%) Indian participants respectively. The mean [standard deviation (SD)] age was 46.3 (10.6) years in the Prucalopride group and 49.9 (6.8) years in the Lactulose group. There were equal amount of male and female patients in the Prucalopride group whereby there were a total of 14 (50%) patients for both male and female gender. There were a total of 33 (56.9%) of male patients and 25 (43.1%) of female patients from the Lactulose group. Majority of the patients were single whereby all the patients in the Lactulose group were single and 92.6% in the Prucalopride group were single. Only 2 patients (7.1%) were married in the Prucalopride group. The majority of the patients were unemployed (92.9%) in the Prucalopride group and all the patients were unemployed in the Lactulose group.

In terms of the patient characteristics, there were no significant differences in terms of the mean Clozapine dose, concomitant use of other antipsychotics and fruits and vegetables intake per day.

The mean Clozapine dose in both the treatment groups was almost similar. The mean (SD) dose of Clozapine in the Prucalopride group was 341.9 (155.9) while the mean dose of Clozapine dose in the Lactulose group was 351.7 (96.3). The minimum dose of Clozapine is 100 mg and the maximum dose of Clozapine is 800 mg in the Prucalopride group. The minimum dose of Clozapine is 100 mg and the maximum dose of Clozapine is 500 mg in the Lactulose group.

Apart from that, the types of concomitant antipsychotics were also similar in both the treatment groups. The concomitant antipsychotics include oral antipsychotics: Haloperidol, Chlopromazine, Aripiprazole, Amisulpiride, Asenapine, Risperidone and depot injection which includes: IM Flupentixol and IM Maintenna. The use of concomitant antipsychotic does have effects on the severity of constipation as other antipsychotics have anticholinergic effect which contributes to constipation.

Apart from antipsychotics, some of the patients in both groups were also prescribed with antidepressants. There were only 2 patients from the Prucalopride group and 1 patient from the Lactulose group who were on antidepressants. All 3 of them were prescribed with antidepressants from the selective serotonin reuptake inhibitor (SSRI) class. Statistical analysis was not done as it is likely not to give any statistical significance due to the small number in both the treatment groups.

### 3.2. Efficacy

Overall, the proportion of patients with ≥3 spontaneous complete bowel movement (SCBM) over the 4 weeks treatment period was higher in the Prucalopride group (85.7%) than in the Lactulose group (60%), reaching significance at Week 4 (*p* = 0.029).

Using the Chi Square test, there was no significant difference between the treatment groups in terms of bowel movement at Week 1 with (*p*-value = 0.360). However, there was a significant difference between the treatment groups in terms of bowel movement at Week 4 with (*p*-value = 0.029). The effect size was large (Φ = 0.63) at the end of the 4 weeks intervention (Table 2).

**Figure 2:**
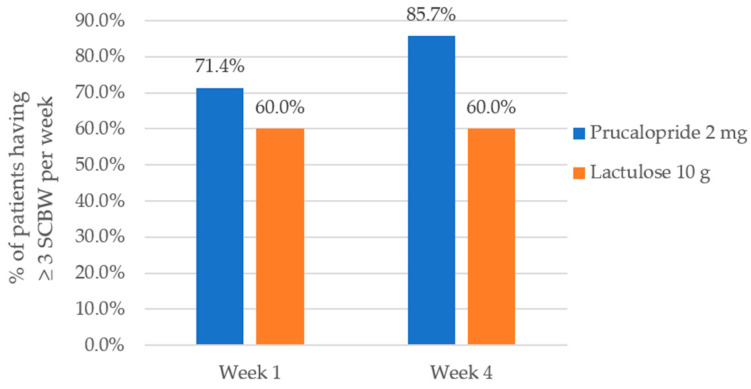
Effect of treatment on the primary endpoint (efficacy). Proportion of patients with ≥3 spontaneous complete bowel movement (SCBM) per week at Week 1 and Week 4, in patients treated with Prucalopride 2 mg and Lactulose 10 g. *p* = 0.360 (χ^2^ value = 0.837) at Week 1 and *p* = 0.029 (χ^2^ value = 4.794) at Week 4.

During Week 1, the proportion of patients treated with Prucalopride 2 mg with ≥3 spontaneous complete bowel movement (SCBM) was 71.4% and there was an increase in efficacy over time whereby the proportion increased to 85.7% during the assessment at the end of Week 4 (Figure 2).

Meanwhile the proportion of patients treated with Lactulose 10 g with ≥3 spontaneous complete bowel movement (SCBM) was 60% at Week 1 and the proportion remained at 60% during assessment at the end of Week 4.

### 3.3. Quality of Life Associated with Constipation

The PAC-QOL assessment was categorized into dissatisfaction subscales which comprise of subscales 1–3, the total score for subscales 1–3 and the treatment satisfaction subscale which is the subscale 4. The analysis was done with the Mann–Whitney U test as presented in Table 3 and Table 4. Overall there was improvement in all four subscales (physical discomfort, psychosocial discomfort, worries and concerns, and treatment satisfaction) for both the treatment groups. However, the mean reduction for the dissatisfaction subscales from baseline to Week 4 were greater in patients treated with Prucalopride 2 mg compared to Lactulose 10 g with statistically significant difference as presented in Table 4. The mean reduction for the treatment satisfaction subscale from baseline to Week 4 was greater in patients treated with Prucalopride compared to Lactulose although there was no statistical significance in comparison.

**Figure 3:**
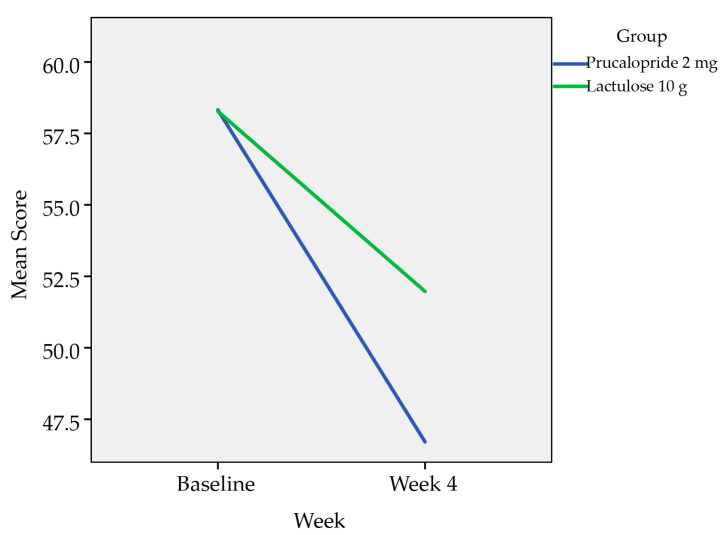
Repeated Measure ANOVA comparing the dissatisfaction subscales (subscales 1–3) from baseline to Week 4.

Repeated measure ANOVA with Huynh–Feldt correction determined that there is a statistically significant difference in the mean subscales 1–3 (dissatisfaction subscales) at baseline and at four weeks between Prucalopride 2 mg and Lactulose 10 g with *p*-value < 0.001. Patients on Prucalopride 2 mg had a significantly larger drop in subscales 1–3 compared to patients on Lactulose 10 g (Figure 3).

**Figure 4:**
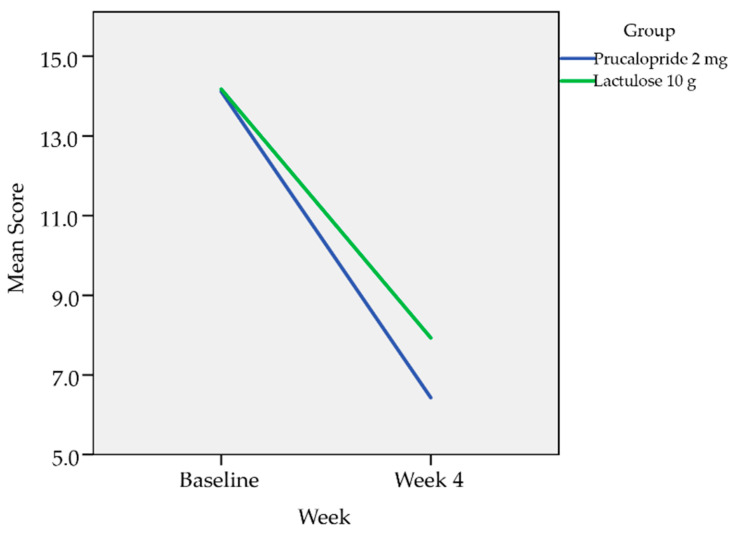
Repeated measure ANOVA comparing the treatment satisfaction subscale (subscale 4) from baseline to Week 4.

Repeated measure ANOVA with Huynh–Feldt correction determined that there is no statistically significant difference in the mean subscale 4 (treatment satisfaction subscale) at baseline and at four weeks between Prucalopride 2 mg and Lactulose 10 g with *p*-value of 0.356. However, the mean reduction was greater in patients treated with Prucalopride compared to Lactulose (Figure 4).

### 3.4. Safety Evaluation: Treatment Emergent Adverse Events

The treatment emergent adverse events in patients who completed the research were summarized in Table 5. The incidence of adverse events due to the treatment was 5/28 (17.8%) in the Prucalopride 2 mg group and 3/30 (10%) in the Lactulose 10 g group. Using the Chi square test, there is no significant difference in treatment emergent adverse events between the treatment groups with *p*-value = 0.386.

Complaints of abdominal pain was higher in those assigned to the Prucalopride 2 mg group (4/28, 14.2%) compared to Lactulose group (1/30, 3.3%). Diarrhea was reported in the Lactulose 10 g group (2/30, 6.7%). There was no adverse event of diarrhea in the Prucalopride 2 mg group. However, loose stools were reported in 1 patient (3.6%) in the Prucalopride 2 mg group.

The presence of mild to moderate abdominal pain in the Prucalopride 2 mg group was reported to occur only during the first week of treatment. This symptom subsided after the first few days of treatment. The similar incident of mild abdominal pain was reported in the Lactulose 10 g group.

There were 2 patients in the Prucalopride 2 mg group who did not complete the study due to treatment emergent adverse events. One patient developed headache for a duration of 4 days and 1 patient reported moderate abdominal pain. In the Lactulose 10 g group, 1 patient discontinued the treatment due to diarrhea. There were no serious treatment emergent adverse events reported in both Prucalopride 2 mg and Lactulose 10 g group. There were no fatalities reported in both the groups.

### 3.5. Other Safety Parameters: Laboratory Investigation and Vital Signs

The differences between the treatment groups (Prucalopride 2 mg and Lactulose 10 g) in terms of other safety parameters which include vital signs (temperature, heart rate, blood pressure and respiratory rate) as well as the laboratory investigation (full blood count and renal profile) were not clinically significant (these data are not shown).

## 4. Discussion

The use of antipsychotics has risen over the years with the widespread of mental health awareness, however, the use of antipsychotics come with several unpleasant consequences due to its adverse effect profiles. Nevertheless, it remains as the savior for patients with psychiatric illness. Due to the prominent anticholinergic effects of Clozapine in comparison to other conventional and non-conventional antipsychotics, special attention should be given to address the issue of gastrointestinal hypomotility in those prescribed with Clozapine to avoid the occurrence of paralytic ileus or other complications associated with prolonged constipation. Clozapine has a receptor profile and mechanism of action that contributes to the occurrence of intestinal hypomotility through anticholinergic activity at the muscarinic receptors (particularly M3), antagonism of 5HT3 and 5HT4 receptors (and, to a lesser extent, 5HT2, 5HT6, and 5HT7) and mild antagonism of the D2 receptors.

First line treatment for Clozapine induced constipation recommended by the American College of Gastroenterology is the use of stool softeners, followed by osmotic laxatives and finally the stimulant laxatives. However, 20–50% of patients who were treated with the first line agents continue to have symptomatic constipation. There have been promising results from the use of newer forms of treatment which are the Linaclotide & Lubiprostone (intestinal secretogogues) and Prucalopride (serotonergic motility medication) as these medications are effective in patients who failed to respond to the first line agents. However, the data is still limited at this current point of time [13].

This current study is the first study in Asia to evaluate the potential efficacy and safety of Prucalopride in treating individuals with constipation secondary to Clozapine.

Prucalopride is a dihydrobenzofurancarboxamide derivative of the benzofurane class. It is an enterokinetic compound which has an exorbitant binding property to 5-HT4 receptors. In other words, it is a 5-HT4 receptor agonist. Acetylcholine is excreted upon activation of the 5-HT4 receptors in the gastrointestinal system. The excretion of acetylcholine which is the excitatory neurotransmitter would lead to motility of the gastrointestinal tract.

There have been several meta-analyses and systematic review evaluating the use of Prucalopride in managing functional constipation compared to placebo in general population. These studies demonstrate the effectiveness of Prucalopride in treating functional constipation compared to placebo with frequently transient but negligible adverse events [14]. Individual double blind placebo control clinical trial studies also demonstrate the effectiveness of Prucalopride compared to placebo in patients with functional constipation [15,16,17]. Up to date, there is no study conducted in Asia to evaluate the use of Prucalopride in Clozapine induced constipation. However, the results of this current study are in keeping with the findings which demonstrate the effectiveness of Prucalopride use in the general population with functional constipation.

The results of this 4 weeks, open label, head to head comparison study between Prucalopride and Lactulose for Clozapine induced constipation in patients with treatment resistant schizophrenia, demonstrates that Prucalopride 2 mg is clinically more efficacious in treating constipation compared to Lactulose 10 g although both the treatment group did denote a change from baseline. In this study, Prucalopride was more effective in the management of clozapine induced constipation than Lactulose, over a four week period. The difference appeared to be of large effect size (Φ = 0.63). While the number of patients studied was substantial given the restricted clinical use of clozapine, it would be helpful to perform randomized blinded trials or a larger prospective cohort as a confirmatory study to further substantiate the findings.

The number needed to treat (NNT) for Prucalopride was 4 during evaluation at Week 4. Subjects in both the treatment group did not require any rescue medication throughout the 4 weeks duration of study. Overall the proportion of patients with a mean frequency on average of ≥3 spontaneous complete bowel movement (SCBM) over the 4 weeks treatment period was higher in the Prucalopride 2 mg group (85.7%) than in the Lactulose 10 g group (60%), reaching significance at Week 4 with a *p*-value = 0.029.

This study shows that a daily dose of Prucalopride 2 mg is effective in treating constipation secondary to Clozapine use. The majority of the patients start to have spontaneous complete bowel movement after Day 3 of Prucalopride 2 mg initiation. This could be a reason to suggest why there was no statistical difference during Week 1 between Prucalopride 2 mg and Lactulose 10 g as these patients only start to have regular bowel movements after Day 3. However, once those prescribed with Prucalopride began to have spontaneous complete bowel movement, they demonstrate a regular pattern of bowel movement during the next 3 weeks.

The proportion of patient with ≥3 spontaneous bowel movement (SCBM) at Week 1 was 71.4% in Prucalopride 2 mg group and 60% in the Lactulose 10 g group. The proportion of patient with ≥3 spontaneous bowel movement (SCBM) at Week 4 was 85.7% in Prucalopride 2 mg group and 60% in the Lactulose 10 g group.

The outcome shows a steady increase in efficacy from Week 1 to Week 4 in patients prescribed with Prucalopride 2 mg. The majority of those prescribed with Lactulose 10 g did not seem to have increment in spontaneous complete bowel movement during the next 3 weeks as the proportion was plateau at 60% during the assessment at Week 4.

The PAC-QOL assessment was carried out at baseline and Week 4. In this study, the assessment was categorized into dissatisfaction subscales which comprise of subscales 1–3, the total score for subscales 1–3 and the treatment satisfaction subscale which is the subscale 4. The analysis was done with the Mann–Whitney U test. Overall, there was improvement in all four subscales for both the treatment groups. However, the mean reduction for the dissatisfaction subscales from baseline to Week 4 were greater in patients treated with Prucalopride 2 mg compared to Lactulose 10 g with statistically significant difference. The mean reduction for the satisfaction subscale from baseline to Week 4 was greater in patients treated with Prucalopride 2 mg compared to Lactulose10 g although there was no statistically significant difference in comparison.

Complaints of abdominal pain was present in both the treatment group however the incident was slightly higher in the Prucalopride 2 mg group compared to the Lactulose 10 g group. The other complaints in the Prucalopride 2 mg group were presence of loose stools and one participant who did not complete the study had complained of headache. In the Lactulose group, there were complaints of diarrhea. There was no significant difference in treatment emergent adverse events between the treatment groups with *p*-value = 0.386.

Constipation is a common adverse effect of Clozapine. Although there are no established guidelines to manage this condition, regular monitoring should be ensured in order to prevent complications of constipation. The patient should be informed regarding the possible adverse effects. Equal importance should be given to constipation similarly to other adverse effects associated with Clozapine such as seizures, agranulocytosis and myocarditis. Patients should also be encouraged to consume more fiber and increase their fluid intake.

There have not been many literatures on this topic and there is no data to compare this study as this is the first pilot study conducted to evaluate the efficacy and safety of Prucalopride versus Lactulose in constipation secondary to Clozapine use in Asia. There was a limited number of patients who were solely on Clozapine for treatment resistant schizophrenia in University Malaya Medical Centre. The majority of them were on a combination treatment with other antipsychotic medication. Hence, patients on other antipsychotics apart from Clozapine were included in this study. However, there was no significant difference in both the treatment groups when comparison was done on the SCBM in participants who were solely on Clozapine and on concomitant AP in both the treatment groups. (data not shown)

The use of Prucalopride can be considered in patients with constipation secondary to Clozapine use who fails to respond to other conventional laxatives. From this study, Prucalopride is effective and safe in treating constipation induced by Clozapine use.

## 5. Conclusions

The conclusions that can be drawn from this study are that Prucalopride was more efficacious than Lactulose in the management of Clozapine induced constipation, over a 4 week period. The number needed to treat (NNT) for Prucalopride was 4 during evaluation at the end of this 4 weeks interventional study. The use of Prucalopride can be taken into consideration as a treatment of antipsychotic related constipation in patients who do not improve with conventional laxatives. There have been emergences of adverse effects reported with the use of Prucalopride in this study however these adverse effects are transient and subsides over the first few days. This study also shows that the use of Prucalopride improves the quality of life associated with constipation.

## Figures and Tables

**Table 1:** Demographics and characteristics of participants with clozapine induced constipation.

Characteristic	Prucalopride 2 mg (*N* = 28)	Lactulose 10 g (*N* = 30)	χ^2^ Value	*p*-Value
**Age (years)**				
Mean (SD)	46.3 (10.6)	49.9 (6.8)		0.12
**Gender, *n* (%)**				
Men	14 (50)	19 (63)	1.05	0.306
Women	14 (50)	11 (36.6)		
**Ethnicity, *n* (%)**				
Malay	4 (14.3)	7 (23.3)	0.776	0.678
Chinese	20 (71.4)	19 (63.3)		
Indian	4 (14.3)	4 (13.3)		
**Marital Status**				
Single	26 (92.9)	30 (100)	2.219	0.136
Married	2 (7.1)	0 (0)		
**Employment, *n* (%)**				
Unemployed	27 (92.9)	30 (100)	1.09	0.296
Employed	1 (3.6)	0 (0)		
**Fruits & Vegetables intake per day**				
5 servings	3 (10.7)	5 (16.6)	0.076	0.783
<5 servings	25 (89.2)	25 (83.3)		
**Concomitant use of other Antipsychotics, *n* (%)**				
Yes	14 (50)	12 (40)	0.586	
No	14 (50)	18 (60)		0.4444
**Clozapine Dose**				
Mean (SD)	341.9 (155.9)	351.7 (96.3)		0.775

**Table 2:** Frequency of bowel movement during Week 1 and at the end of the 4 weeks intervention.

Frequency of Bowel Movement	Prucalopride 2 mg		Lactulose 10 g		Chi Square	*p*-Value	Effect Size (Φ)
	Mean ± SD	Median	Mean ± SD	Median			
**Week 1**	2.96 (0.744)	3.00	2.83 (1.234)	3.00	0.837	0.360	0.11
**Week 4**	4.93 (1.884)	4.50	3.67 (2.057)	3.00	4.794	0.029	0.63

**Table 3:** Patient assessment of constipation—quality of life (PAC-QOL) assessment at baseline.

Baseline	Prucalopride, *N* = 28		Lactulose, *N* = 30		Man Whitney Test	*p*-Value	Effect Size
	Mean ± SD	Median	Mean ± SD	Median			
**Subscale 1** (Physical discomfort)	10.82 (0.819)	11.0	10.83 (0.834)	11.0	−0.042	0.967	0.01
**Subscale 2** (Psychological discomfort)	21.71 (0.810)	21.5	21.56 (0.802)	21.0	−0.239	0.811	0.03
**Subscale 3** (Worries and concerns)	25.79 (0.783)	26.0	25.77 (0.728)	26.0	−0.093	0.926	0.01
**Total** (Subscales 1–3)	58.32 (1.188)	59.0	58.27 (1.230)	59.0	−0.138	0.890	0.02
**Subscale 4** (Treatment satisfaction)	14.11 (0.786)	14.0	14.17 (0.747)	14.0	−0.275	0.783	0.04

**Table 4:** PAC-QOL assessment at the end of the 4 weeks study intervention.

Week 4	Prucalopride, *N* = 28		Lactulose, *N* = 30		Man Whitney Test	*p*-Value	Effect Size
	Mean ± SD	Median	Mean ± SD	Median			
**Subscale 1** (Physical discomfort)	7.11 (1.892)	7.0	8.70 (1.622)	8.0	−3.385	0.001	0.44
**Subscale 2** (Psychological discomfort)	17.32 (1.926)	17.0	19.63 (1.542)	19.0	−4.760	<0.001	0.63
**Subscale 3** (Worries and concerns)	22.29 (1.462)	22.0	23.62 (2.092)	23.0	−2.489	0.013	0.33
**Total** (Subscales 1–3)	46.71 (4.224)	46.0	51.97 (4.679)	51.0	−5.140	<0.001	0.67
**Subscale 4** (Treatment satisfaction)	6.43 (3.756)	5.0	7.93 (4.433)	6.0	−0.924	0.356	0.12

**Table 5:** Treatment emergent adverse events.

**Adverse Event, *n* (%)** **(Completed Treatment)**	**Prucalopride 2 mg** **(*N* = 28)**	**Lactulose 10 g** **(*N* = 30)**
Abdominal pain	4 (14.2)	1 (3.3)
Diarrhea	0	2 (6.7)
Loose stool	1 (3.6)	0
Total	5 (17.8)	3 (20)
**Adverse Event, *n*** **(did not completed treatment)**		
Headache	1	0
Abdominal pain	1	0
Diarrhea	0	1
